# Human Retinal Organoid Model of Ocular Toxoplasmosis

**DOI:** 10.3390/pathogens14030286

**Published:** 2025-03-14

**Authors:** Liam M. Ashander, Grace E. Lidgerwood, Amanda L. Lumsden, João M. Furtado, Alice Pébay, Justine R. Smith

**Affiliations:** 1 Flinders Health and Medical Research Institute, College of Medicine and Public Health, Flinders University, Adelaide, SA 5042, Australia; liam.ashander@flinders.edu.au (L.M.A.); amanda.lumsden@unisa.edu.au (A.L.L.); 2 Department of Anatomy and Physiology, The University of Melbourne, Parkville, VIC 3010, Australia; grace.lidgerwood@unimelb.edu.au (G.E.L.); apebay@unimelb.edu.au (A.P.); 3 Division of Ophthalmology, Ribeirão Preto Medical School, University of São Paulo, Ribeirão Preto 14049-900, São Paulo, Brazil; furtadojm@gmail.com; 4 Department of Surgery, Royal Melbourne Hospital, The University of Melbourne, Parkville, VIC 3052, Australia

**Keywords:** uveitis, retinitis, ocular toxoplasmosis

## Abstract

The health burden of ocular toxoplasmosis is substantial, and there is an unmet need for safe and curative anti-microbial drugs. One major barrier to research on new therapeutics is the lack of in vitro human-based models beyond two-dimensional cultured cells and tissue explants. We aimed to address this research gap by establishing a human retinal organoid model of ocular toxoplasmosis. Retinal organoids, generated from human induced pluripotent stem cells and grown to two stages of organization, were incubated with a suspension of live or heat-killed GT-1 strain *T. gondii* tachyzoites, or medium without tachyzoites. Both developing (1 month post-isolation) and matured (6 months post-isolation) organoids were susceptible to infection. Spread of live parasites from the margin to the entire organoid over 1 week was indicated by immunolabelling for *T. gondii* surface antigen 1. This progression was accompanied by changes in the levels of selected tachyzoite transcripts—*SAG1*, *GRA6*, and *ROP16*—and human cytokine transcripts—*CCL2*, *CXCL8*, *CXCL10*, and *IL6*—in infected versus control conditions. Our human retinal organoid model of ocular toxoplasmosis offers the opportunity for many future lines of study, including tachyzoite interactions with retinal cell populations and leukocyte subsets, parasite stage progression, and disease processes of different *T. gondii* strains, as well as drug testing.

## 1. Introduction

*Toxoplasma gondii* is an Apicomplexan protozoan parasite that infects an estimated one-third of the global population [1]. Ocular toxoplasmosis is the most frequent clinical presentation of the infection [2], with a prevalence that ranges quite widely across world regions, from 0.7% to 5.8% [3,4]. In healthy adults and children, ocular toxoplasmosis is characterized by recurrent unilateral necrotizing retinal inflammation [5]. Large cohort studies indicate that there is severe vision impairment in approximately one-quarter of affected eyes [6,7]. Congenital ocular toxoplasmosis is more likely to result in sight-threatening disease, including bilateral eye involvement, and large and central retinal lesions [8], and while early treatment can improve the outcomes, long-term consequences still may be severe [9]. Multiple antimicrobial drugs are used singly or in combination, along with corticosteroids, to treat an attack of toxoplasmic retinal inflammation [10]. None of these antimicrobial drugs are curative of an infection in the patient, and several are associated with serious systemic toxicity [5]. In essence, the health burden of ocular toxoplasmosis is substantial, and there is a clear unmet need for safe and curative anti-microbial drugs.

One major barrier to research on new therapeutics for ocular toxoplasmosis is the lack of in vitro human-based models beyond two-dimensional cultured cells and tissue explants [11,12]. Dissociated retinal cultures prepared from fetal or adult retina preserve many retinal cell types, but lack the organization seen in the intact retina, and the cellular composition may change markedly as the culture proliferates [13]. Cultured retinal explants preserve the complex architecture of the retina, but the system relies on access to cadaver donor eyes shortly after death, and tissue structure may be lost in long-term culture [14]. Retinal organoids are self-organizing, and undergo spatial and time-restricted lineage commitments similar to those seen in vivo, and hence they can recapitulate cell subpopulations, tissue architecture, and even some functions [15]. Organoids generated from either embryonic stem cells (ESCs) or induced pluripotent stem cells (iPSCs) undergo a process of maturation in extended culture that is similar to normal development, and thus also offer the opportunity to study the retina at different pre- and post- natal stages [16,17]. Single-cell RNA sequencing of iPSC-derived retinal organoids at several time-intervals across development shows substantial agreement between the transcriptomic profiles of early-harvest organoids and the developing retina, and matured organoids and adult retinal explants [18,19].

Seeking to establish an improved model for experimental studies of ocular toxoplasmosis, we took advantage of the recent developments in retinal organoid science. In this article, we describe the infection of retinal organoids, generated from iPSCs and grown to two stages of organization, with natural strain *T. gondii* tachyzoites. Immunohistochemistry and reverse transcription-quantitative polymerase chain reaction (RT-qPCR) were used to characterize the progression of, and response to, *T. gondii* infection in this new human retinal organoid model of ocular toxoplasmosis.

## 2. Materials and Methods

### 2.1. Toxoplasma gondii

GT-1 strain *Toxoplasma gondii* (gift of Jitender P. Dubey, PhD, US Department of Agriculture, Beltsville, Maryland; and L. David Sibley, PhD, Washington University, St. Louis, MO) was cultured in tachyzoite form by serial passage in human neonatal dermal fibroblast monolayers (Thermo Fisher Scientific-Cascade Biologics Invitrogen, Carlsbad, CA, USA), typically every 2 days. Human dermal fibroblasts were maintained at 37 °C and 5% CO_2_ in air, in Dulbecco’s Modified Eagle Medium (DMEM) supplemented with 10% heat-inactivated fetal bovine serum (FBS) (both from Thermo Fisher Scientific-Gibco, Grand Island, NY, USA: catalogue numbers 11965 and 10099, respectively). For *T. gondii* culture, the FBS supplement in the DMEM was reduced from 10% to 1%.

### 2.2. Human Retinal Organoids

Retinal organoids were generated from human skin-derived iPSCs, according to previously published methods [17,20]. In brief, human iPSCs (WAB220) were cultured on plates pre-coated with 10 μg/mL Vitronectin XF substrate, prepared in CellAdhere Dilution Buffer (both from STEMCELL Technologies, Vancouver, Canada), in StemFlex medium supplemented with 10% StemFlex Supplement (both from Thermo Fisher Scientific-Gibco), refreshed every second day. Cell colonies were maintained by weekly passage using ReLeSR Passaging Reagent (STEMCELL Technologies). To generate the retinal organoids, iPSCs at 70–80% confluence were switched into Essential 6 medium (Thermo Fisher Scientific-Gibco, catalogue number A1516401) for 2 days, and subsequently into Essential 6 medium containing 1% CTS N-2 supplement, 10 U/mL penicillin, and 10 µg/mL streptomycin (all from Thermo Fisher Scientific-Gibco). After 4 weeks, formed retinal organoids were isolated with a needle, and cultured at 37 °C and 5% CO_2_ in air in low-attachment, 6-well plates (9.5 cm^2^ area/well) containing complete organoid medium (with all components from Thermo Fisher Scientific-Gibco): DMEM/F-12 (catalogue number 11330), 1% MEM non-essential amino acids solution, 2% B-27 supplement, 10 U/mL penicillin, and 10 µg/mL streptomycin, supplemented with 10 ng/mL human recombinant fibroblast growth factor (FGF)-2 (Merck-Sigma Aldrich, St. Louis, MO, USA). Organoids were fed thrice weekly by removing a one-half volume of medium from each well and replacing this with fresh medium. The FGF-2 supplementation of the medium was ceased at 7 days post-isolation, and retinal organoids were cultured in complete organoid medium alone up to week 26. Retinal organoids were approximately 1 mm across at the time of use in infection studies.

### 2.3. Toxoplasma gondii Infection of Retinal Organoids

At approximately 1 or 6 months after isolation, retinal organoids were incubated at 37 °C and 5% CO_2_ in air with a suspension of live or heat-killed tachyzoites in complete organoid medium, or medium containing no tachyzoites, in groups of 2–4 organoids in low-attachment, 12-well plates (3.8 cm^2^ area/well). Freshly egressed tachyzoites were pelleted by centrifugation at 400× *g* for 10 min and resuspended in complete organoid medium at 4 × 10^6^ tachyzoites in 250 µL for addition to individual wells. After 6 to 8 h of organoid–tachyzoite co-culture, the volume of medium in each well was topped up to 1 mL, and the plates were returned to incubation. Groups of retinal organoids were taken 1 day later and then every 2 days up to 7 days of co-culture, and either collected in Trizol Reagent (Thermo Fisher Scientific-Ambion Invitrogen, Carlsbad, CA, USA) and immediately frozen at −80 °C for RNA extraction and RT-qPCR for selected human cell and parasite transcripts listed in Table 1, or fixed for 3 h in 10% neutral buffered formalin and stored in phosphate-buffered saline at 4 °C for embedding and immunolabelling for parasite antigen. The medium was refreshed at 3 and 5 days if applicable, with removal and replacement of 500 µL volumes of complete organoid medium. Plaque assays were performed in parallel with the organoid–tachyzoite co-cultures to confirm the viability of live tachyzoites, and the effectiveness of the heat-killing (at 55 °C for 30 min).

### 2.4. Immunohistochemistry

Retinal organoids were dehydrated from PBS by passage through graded ethanol solutions and cleared in chloroform. They then were moved through two changes of Surgipath Paraplast wax (Leica Microosystems, North Ryde, Australia) with no vacuum, and finally embedded. The embedded organoids were sectioned at 10 microns thickness and mounted on Superfrost glass slides. Serial sections from the middle one-third of each organoid were deparaffinized in xylene, followed by graded ethanol solutions, and lastly deionized water. After blocking in 2% normal goat serum (Vector Labs, Burlingame, CA, USA) in PBS for 30 min at room temperature, the organoid sections were incubated overnight at 4 °C with mouse monoclonal IgG2a anti-*T. gondii* surface antigen 1 (SAG1) antibody (clone D61S; Thermo Fisher Scientific-Invitrogen, Rockford, IL, USA) or isotype-matched negative control antibody (clone C1.18.4; BD Pharmingen, San Jose, CA, USA), both diluted to 2 µg/mL in blocking solution. Subsequently, sections were washed in PBS and incubated for 30 min with biotinylated goat anti-mouse IgG antibody (Vector Labs) at 10 µg/mL in blocking solution, followed by 45 min with Vectastain ABC Kit (Vector Labs) diluted 100-fold in tris-buffered saline with 0.01% Triton X-100 at room temperature. Antibody complexes were identified using HighDef Red IHC chromogen (AP) reagent (Enzo Scientific, Farmingdale, NY, USA) plus Hematoxylin QS Counterstain (Vector Labs). All organoid sections were covered with ProLong Diamond Antifade Mountant (Thermo Fisher Scientific-Molecular Probes, Eugene, OR, USA) and imaged by light microscopy under oil at 1000-times magnification.

### 2.5. RNA Extraction and Reverse Transcription

Total RNA was extracted by Trizol reagent according to the manufacturer’s instructions. RNase-free glycogen was added to aid precipitation of RNA pellets. The pellets were resuspended in 20–30 µL of nuclease-free water and heated to 55 °C for 10 min prior to determination of nucleic acid concentration on the Nanodrop 2000 spectrophotometer (Thermo Fisher Scientific, Wilmington, DE, USA). RNA yield across the organoids ranged 198–1368 ng (median = 831 ng). Extracted RNA was frozen at −80 °C prior to cDNA synthesis. cDNA was synthesized using iScript Reverse Transcription Supermix for RT-qPCR (Bio-Rad, Hercules, CA, USA) with 75–150 ng of RNA per reaction. Two identical reactions were run for each sample and subsequently pooled. cDNA was stored frozen at −20 °C prior to qPCR.

### 2.6. Quantitative Polymerase Chain Reaction

The qPCR was performed on the CFX Connect Real-Time PCR Detection System (Bio-Rad), using 2 µL of cDNA, 4–10 µL of iQ SYBR Green Supermix, or SsoAdvanced SYBR Green Supermix (Bio-Rad), and 0.3–1.5 µL each of 10 µM forward and reverse primers, plus nuclease-free water for each reaction. The amplification consisted of the following: a pre-cycling hold at 95 °C for 5 min; 40 cycles of denaturation for 30 s at 95 °C; annealing for 30 s at 58 °C, 60 °C, or 62 °C; extension for 30 s at 72 °C; and a post-extension hold at 75 °C for 1 s. A melting curve, representing a 1 s hold at every 0.5 °C between 70 °C and 95 °C, was generated to confirm that a single peak was produced for each primer set. Primer efficiency for all pairs was 80% or greater. The amplicon size of each PCR product was confirmed by electrophoresis on 2% agarose gel. The cycle threshold was measured with Cq determination mode set to regression, and relative expression was normalized to the geometric mean of two stable reference genes, ribosomal lateral stalk protein subunit P0 (*RPLP0*) and tyrosine 3-monooxygenase/tryptophan 5-monooxygenase activation protein zeta (*YWHAZ*). Table 2 shows primer pair sequences and amplicon sizes.

### 2.7. Statistical Analysis

Data were analyzed using GraphPad Prism v9.0.0 (GraphPad Software, Boston, MA, USA). Comparisons between two conditions were made using an unpaired two-tailed *t*-test. Welch’s correction was applied in comparisons for which an F test indicated variances were unequal. Two-way ANOVA (main effects model) with the Tukey test was utilized to account for multiple variables simultaneously. Area under the curve (AUC) was calculated with the AUC analysis tool, and comparisons were made by an unpaired two-tailed *t*-test. A statistically significant difference was defined as *p* < 0.05 for all comparisons. Multidimensional scaling was performed in R v4.4.1 using the plotMDS function of R package limma v3.60.4.

## 3. Results

**Figure 1:**
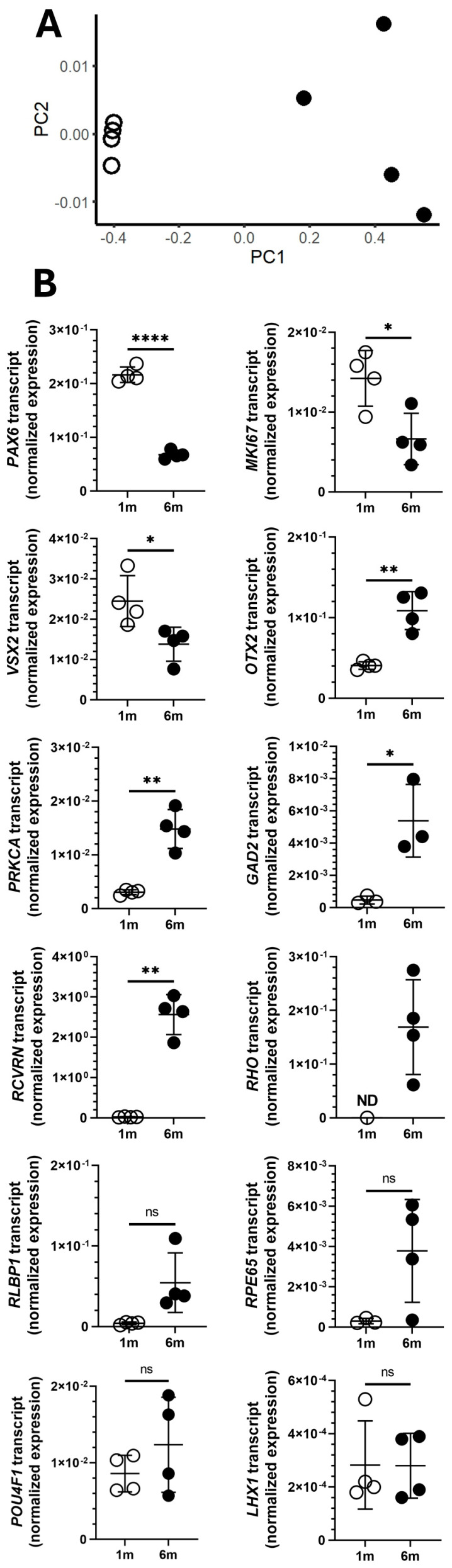
Expression of proliferation, multipotency, or retinal cell-specific gene transcripts in human retinal organoids. Expression levels of 12 transcripts were measured by quantitative polymerase chain reaction and normalized to two stable reference genes (*RPLP0* = ribosomal lateral stalk protein subunit P0 and *YWHAZ* = tyrosine 3-monooxygenase/tryptophan 5-monooxygenase activation protein zeta). In all visualizations, open and closed circles represent gene transcript expression in 1-month and 6-month (m) organoids, respectively (*n* = 4 organoids/time interval, cultured in different wells of 12-well plates). (**A**) Multi-dimensional scaling plot showing distance relationships within and between retinal organoid groups, based on normalized expression of the 12 gene transcripts. (**B**) Graphs presenting expression of individual transcripts. Transcript expression levels were compared by unpaired two-tailed *t*-test with Welch’s correction: cross bar = mean expression; error bar = standard deviation; ns = not significant; * = *p* < 0.05; ** = *p* < 0.01; **** = *p* < 0.0001. ND = transcript not detectable ≥ 3 of 4 organoids. Labels: *PAX6* = paired box 6, *MKI67* = marker of proliferation Ki-67, *VSX2* = visual system homeobox 2, *OTX2* = orthodenticle homeobox 2, *POU4F1* = POU class 4 homeobox 1, *LHX1* = LIM homeobox 1, *GAD2* = glutamate decarboxylase 2, *PRKCA* = protein kinase c alpha, *RHO* = rhodopsin, *RCVRN* = recoverin, *RLBP1* = retinaldehyde binding protein 1, and *RPE65* = retinoid isomerohydrolase RPE65. Graphs showing these data as fold-change are presented in Figure S1.

Differences in the expression of 8 of 12 key proliferation, multipotency, and retinal cell-specific markers confirmed the early and mature developmental stages of the retinal organoids that were infected with *T. gondii* at 1 month and 6 months, respectively (Figure 1). Multidimensional scaling analysis for the 12 transcripts demonstrated clear separation between organoids cultured for 1 month versus 6 months, with more variation in gene expression of individual organoids after 6 months of culture (Figure 1A). In comparison to the 1-month organoids, 6-month organoids expressed significantly lower levels of marker of proliferation Ki-67 (*MKI67*), paired box 6 (*PAX6*, progenitor cell marker), and visual system homeobox 2 (*VSX2*, progenitor cell marker) (*p* ≤ 0.031); and significantly higher levels of orthodenticle homeobox 2 (*OTX2*, photoreceptor marker), protein kinase c alpha (*PRKCA*, bipolar cell marker), glutamate decarboxylase 2 (*GAD2*, amacrine cell marker), and recoverin (*RCVRN*, photoreceptor marker) (*p* ≤ 0.019); and presented the only expression of rhodopsin (*RHO*, photoreceptor marker) (Figure 1B). Mean expression levels of retinaldehyde binding protein 1 (*RLBP1*, glial cell marker) and retinoid isomerohydrolase RPE65 (*RPE65*, pigment epithelial cell marker) by 6-month organoids were 13.6-fold and 12.8-fold higher, respectively, than by 1-month organoids, but with considerable variation and thus not statistically significantly different. The expression levels of POU class 4 homeobox 1 (*POU4F1*, ganglion cell marker) and LIM homeobox 1 (*LHX1*, horizontal cell marker) were similar between the two organoid groups.

**Figure 2:**
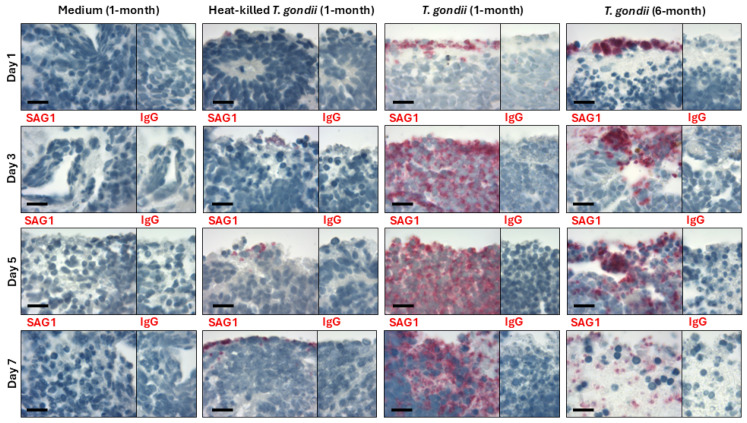
*Toxoplasma gondii* tachyzoite infection of human retinal organoids. Representative photomicrographs of 1-month or 6-month retinal organoids immunolabeled for *T. gondii* surface antigen 1 (SAG1) or species- and isotype-matched immunoglobulin (IgG), taken up to 7 days following incubation with live or heat-killed GT-1 tachyzoites, or medium only. Fast Red chromogen (red) with hematoxylin nuclear counterstain (blue). Original magnification = 1000×. Scale bar = 20 microns. Selected low power magnification photomicrographs and enlarged sections are provided in Figure S2 and Figure S3, respectively.

To investigate the possibility of infecting early and mature retinal organoids with *T. gondii*, the developing 1-month and mature 6-month organoids were incubated in a suspension of live GT-1 strain *T. gondii* tachyzoites for up to 7 days (Figure 2). Controls included similarly aged organoids incubated with heat-killed tachyzoites or complete organoid medium only. Infection was first evaluated by immunohistochemical detection of tachyzoite surface marker, SAG1, in retinal organoid sections. Sections of 1-month and 6-month retinal organoids incubated with T. gondii tachyzoites had positively labelled bodies along and just inside the tissue margins after 1 day of co-culture. At 3 days, labelled bodies could be seen within the tissue. By 5 and 7 days, many labelled bodies were observed throughout the tissue, although the positive labelling was more extensive in the 1-month than the 6-month organoids at both time points. Sections of 1-month and 6-month retinal organoids incubated with heat-killed T. gondii tachyzoites for up to 7 days revealed small numbers of positively stained bodies at the edges of, but never within, the tissue. Sections of organoids that were incubated with medium alone for up to 7 days (i.e., non-infected group), as well as sections of organoids incubated under all conditions and across time points but labelled with isotype-matched negative control primary antibody, showed no positive staining. These results indicate that *T. gondii* tachyzoites are able to invade and replicate within human retinal organoids, and suggest that developing organoids are particularly supportive of the parasitic infection.

**Figure 3:**
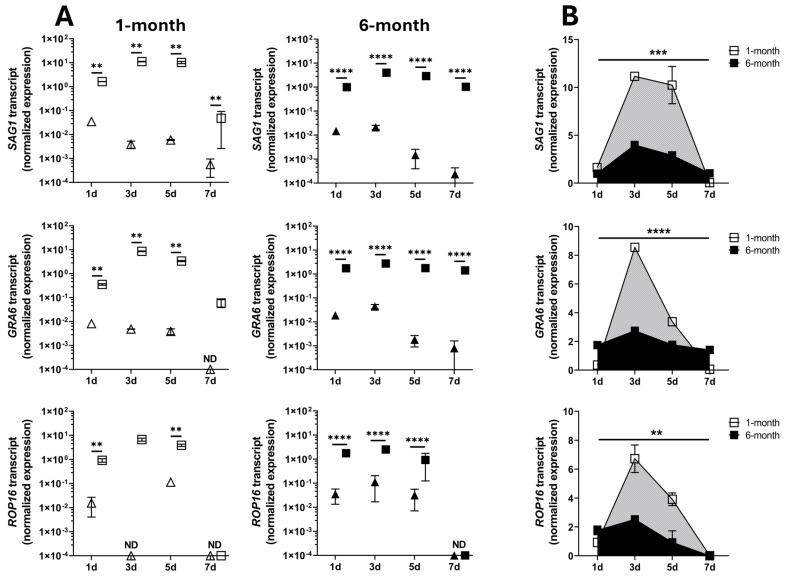
Expression of *T. gondii* tachyzoite gene transcripts in human retinal organoids. After incubation for up to 7 days with live or heat-killed GT-1 tachyzoites, or medium only, expression levels of three transcripts in 1-month or 6-month retinal organoids were measured by quantitative polymerase chain reaction and normalized to two stable reference genes (*RPLP0* = ribosomal lateral stalk protein subunit P0 and *YWHAZ* = tyrosine 3-monooxygenase/tryptophan 5-monooxygenase activation protein zeta). In all visualizations, open and closed symbols represent gene transcript expression in 1-month and 6-month organoids, respectively (*n* = 3 replicates/organoid). Triangles represent organoids incubated with heat-killed tachyzoites, and squares represent organoids incubated with live tachyzoites. *T. gondii* tachyzoite transcripts were not detected in organoids incubated with medium only. Labels: *SAG1* = surface antigen 1, *GRA6* = dense granule protein 6, and *ROP16* = rhoptry protein 16. (**A**) Transcript expression levels were compared by ordinary two-way ANOVA with Tukey’s multiple comparison test and (**B**) areas under the curve were compared by unpaired two-tailed *t*-test: cross bar = mean expression; error bar = standard deviation; ** = *p* < 0.01; *** = *p* < 0.001; **** = *p* < 0.0001. ND = transcript not detectable ≥ 2 of 3 replicates.

The potential for infection of early and mature retinal organoids was also studied by RT-qPCR of genes expressed by *T. gondii* tachyzoites: *SAG1*, dense granule protein 6 (*GRA6*), and rhoptry protein 16 (*ROP16*) (Figure 3). Co-culture of both 1-month and 6-month organoids with live tachyzoites had a highly significant effect on the level of tachyzoite transcripts (*p* ≤ 0.028), with multiple comparison testing showing significant increases in the expression of *SAG1* (*p* ≤ 0.002), *GRA6* (*p* ≤ 0.009), and *ROP16* (*p* ≤ 0.008) genes for all timepoints when comparisons were possible, compared to organoids co-cultured with heat-killed tachyzoites (Figure 3A). As expected, the tachyzoite transcripts were not detected in organoids incubated with complete organoid medium only. These results confirm that *T. gondii* tachyzoites are capable of infecting human retinal organoids. Moreover, an AUC calculation for tachyzoite gene expression in the infected organoids aligned with the immunohistochemistry observation that developing organoids were relatively susceptible to infection: the AUC was significantly greater in 1-month versus 6-month retinal organoids for *SAG1* (*p* = 0.0006), *GRA6* (*p* < 0.0001), and *ROP16* (*p* = 0.002) transcripts (Figure 3B).

**Figure 4:**
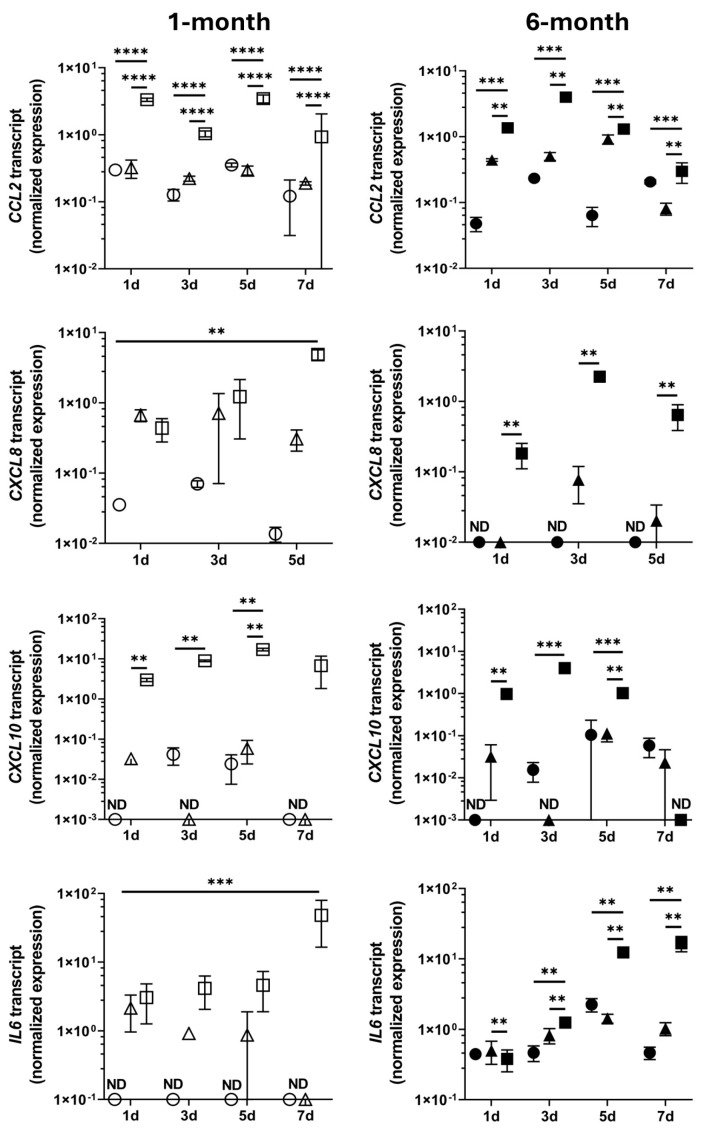
Expression of cytokine gene transcripts in human retinal organoids. After incubation for up to 7 days (d) with live or heat-killed GT-1 *T. gondii* tachyzoites, or medium only, expression levels of four transcripts in 1-month or 6-month retinal organoids were measured by quantitative polymerase chain reaction and normalized to two stable reference genes (*RPLP0* = ribosomal lateral stalk protein subunit P0 and *YWHAZ* = tyrosine 3-monooxygenase/tryptophan 5-monooxygenase activation protein zeta). In all visualizations, open and closed symbols represent gene transcript expression in 1-month and 6-month organoids, respectively (*n* = 3 replicates/organoid). Circles represent organoids incubated with medium only; triangles represent organoids incubated with heat-killed tachyzoites; and squares represent organoids incubated with live tachyzoites. Transcript expression levels were compared by ordinary two-way ANOVA with Tukey’s multiple comparison test: cross bar = mean expression; error bar = standard deviation; ** = *p* < 0.01; *** = *p* < 0.001; **** = *p* < 0.0001. ND = transcript not detectable ≥ 2 of 3 replicates. Labels: *CCL2* = c-c motif chemokine ligand 2, *CXCL8* = c-x-c motif chemokine ligand 8, *CXCL10* = c-x-c motif chemokine ligand 10, and *IL6* = interleukin-6.

The capacity of early and mature retinal organoids to respond to *T. gondii* tachyzoite infection was determined by RT-qPCR for human cytokine transcripts *CCL2*, *CXCL8*, *CXCL10*, and interleukin-6 (*IL6*) (Figure 4). For 1-month and 6-month organoids, tachyzoite infection had a significant effect on the levels of all four transcripts compared to the heat-killed tachyzoite and medium only control conditions (*p* ≤ 0.02). Multiple comparison testing indicated significant up-regulation of *CCL2* transcript at all time points (*p* ≤ 0.006) and of *CXCL10* transcript up to day 5 (*p* ≤ 0.008) for the two organoid groups. In 6-month organoids, but not the 1-month organoids, *CXCL8* transcript was significantly up-regulated up to day 5 (*p* ≤ 0.009), and *IL6* transcript was significantly up-regulated at day 3 through day 7 (*p* ≤ 0.005). These findings indicate that *T. gondii* tachyzoite infection induces a molecular response from retinal organoids which might be more pronounced for the 6-month group than the 1-month group.

## 4. Discussion

As an in vitro human-based model for studying ocular toxoplasmosis, retinal organoids would address several shortcomings of two-dimensional cultured cell and tissue explant models. Through the experimental work presented in this article, we show that developing and matured human retinal organoids are susceptible to infection with *T. gondii* tachyzoites. Some retinal cell marker transcripts showed considerable variation in expression particularly in the matured organoids, indicating a degree of heterogeneity in the developmental stage across those organoids. The spread of the infection from the margin to the entire organoid over 1 week was indicated by immunolabelling for tachyzoite SAG1. This progression was accompanied by significant changes in the levels of selected tachyzoite transcripts—*SAG1*, *GRA6*, and *ROP16*—and human cytokine transcripts—*CCL2*, *CXCL8*, *CXCL10*, and *IL6*—in comparison to heat-killed parasite and medium only control conditions.

Organoid-based models have been developed to elucidate basic processes of multiple infectious conditions, particularly viral diseases. Examples include organoids simulating a spectrum of tissues for COVID-19 research [58], brain organoids for the study of Zika microcephaly [59], and skin and colon organoids for the investigation of Mpox [60,61]. This is the first report of a human retinal organoid model of ocular toxoplasmosis; however, recent publications have described human brain organoid models of *T. gondii* infection. Correa Leite et al. [62] showed that ME49 strain *T. gondii* tachyzoites were capable of invading, and subsequently proliferating and encysting within, human iPSC-derived ‘Brainspheres’. The infection induced changes in the levels of pre-synaptic proteins, cytokines, and growth factors. Seo et al. [63] used ESC-generated human cerebral organoids to track the transformation of ME49 and RH strain *T. gondii* from tachyzoites to bradyzoites by microscopy and transcriptomic sequencing. Studies using intestinal organoids to examine interactions between *T. gondii* tachyzoites and the gut epithelium have also been reported [64].

Single-cell RNA sequencing reveals a high level of agreement in transcriptomic profiles of retinal organoids and fetal retinal tissues at similar time points in development [19]. Our observations indicate that developing retinal organoids may be more supportive of *T. gondii* tachyzoite replication than more mature organoids. Implementing the method previously employed by Chu et al. [65] to study replication of severe acute respiratory syndrome coronaviruses in epithelial cells, we recorded higher *T. gondii* replication in 1-month than 6-month retinal organoids by the AUC of tachyzoite transcript levels. Increased cell proliferation, as evidenced by higher levels of *MKI67* transcript, may contribute to higher parasite replication in developing organoids. Furthermore, *T. gondii* preferentially attaches to and invades host cells in the S-phase [66,67]. Our finding has translational relevance because congenital forms of ocular toxoplasmosis are characterized by relatively extensive retinal destruction [8], consistent with more aggressive infections. In a direct comparison of pre- and postnatally acquired ocular toxoplasmosis, eyes with congenital infections had worse visual acuity and a higher prevalence of legal blindness [68]. Moreover, as highlighted in a recent review of congenital toxoplasmosis by Dubey et al., the severity of this condition is inversely rated to the interval in utero at the time of infection [69]. Thus, we propose that early-harvest retinal organoids, characterized by a high expression of proliferation and multipotency genes and a low expression of retinal cell-specific genes, provide the best option for modelling congenital ocular toxoplasmosis specifically.

In our experiments, retinal organoids infected with *T. gondii* appeared grossly similar by low-power microscopy to those exposed to heat-killed tachyzoites or medium alone, through the 1-week infection period. In contrast, immunohistochemistry showed obvious retinal cell loss in the matured organoid 7 days following *T. gondii* infection. Consistently, RNA yields were lowest in developing and mature organoids infected for 7 days, and *ROP16* transcript was not detectable in infected organoids at this time point, indicating reduced tachyzoite replication. Retinal cell loss will reflect in part direct cell lysis at the time of tachyzoite egress [70]. Infection with *T. gondii* also may induce apoptosis, depending on host cell population and parasite strain [71,72,73]. Additionally, necrosis or apoptosis of non-infected cells may be triggered by inflammatory mediators or other molecules released from adjacent parasite-infected or lysed cells [74].

Our work demonstrates that human retinal organoids have the capacity to respond to *T. gondii* tachyzoites, with developing and matured organoids up-regulating *CCL2* and *CXCL10* transcripts at multiple time points following infection. These two chemokines are rapidly induced in human primary retinal cell populations following tachyzoite infection [55,75], and both have been measured at increased levels in the blood of patients with ocular toxoplasmosis [76,77]. Work in mouse experimental toxoplasmic encephalitis and retinitis has identified essential roles for CCL2 and CXCL10 in the recruitment of myeloid and lymphoid cell subsets into infected tissues [78,79,80,81]. An antibody targeted to CXCL10 reduces the migration of CD4- and CD8-positive T cells into the retina, and promotes intraocular parasite replication and retinal damage in mouse ocular toxoplasmosis, emphasizing the role of CXCL10 in protecting the infected eye [78]. Similar studies using CCL2 blockade in mouse ocular toxoplasmosis have not been reported; however, mice with chronic *T. gondii* encephalitis present decreased brain infiltration of monocytes, neutrophils, and CD4- and CD8-positive T cells, with increased parasite burden, when the animals’ astrocytes are rendered *Ccl2*-deficient [79].

Toxoplasmosis in humans varies in numerous ways from *T. gondii* infection in mice, the common experimental animal, with different routes of infection, pathogen recognition pathways, and cellular responses [82]. Thus, human systems have major importance for studies of basic pathogenic mechanisms. A human retinal organoid model of ocular toxoplasmosis, which we show to be feasible, provides a new opportunity to closely approximate the human disease. Here, we have focused on representing the acute infection, and the current work is limited to one natural isolate strain. However, in addition to studies of new therapeutics for ocular toxoplasmosis, our model offers many potential future lines of investigation. Examples include: tracking parasite stage progression (such as conversion from the dormant bradyzoite to the rapidly replicating, tissue-destructive tachyzoite), observing tachyzoite infections of individual retinal cell populations, elucidating interactions between infected tissue and different leukocyte subsets, comparing disease processes of different *T. gondii* strains, and understanding the pathology of the relatively aggressive retinitis that characterizes congenital ocular toxoplasmosis. Rapid, efficient, reproducible methods mean human retinal organoid culture is generally accessible [83,84], supporting widespread use of this model.

## Figures and Tables

**Table 1:** Selected human cell and parasite transcripts studied by quantitative polymerase chain reactions.

Name	HGNC Symbol	Description	References
Human Cell Transcripts			
Marker of proliferation ki-67	*MKI67*	Nuclear protein expressed in proliferating cells	[21,22,23]
Paired box 6	*PAX6*	Transcription factor involved in maintaining retinal progenitor cell multipotency	[24,25,26]
Visual system homeobox 2	*VSX2*	Transcription factor involved in retinal progenitor identity and proliferation	[27,28]
Orthodenticle homeobox 2	*OTX2*	Transcription factor involved in photoreceptor and retinal pigment epithelium differentiation	[29,30]
POU class 4 homeobox 1	*POU4F1*	Transcription factor expressed in developing and mature retinal ganglion cell subsets	[31,32]
LIM homeobox 1	*LHX1*	Transcription factor expressed in developing and mature horizontal cells	[33,34]
Glutamate decarboxylase 2	*GAD2*	Enzyme involved in gamma-aminobutyric acid synthesis in amacrine cells	[35]
Protein kinase c alpha	*PRKCA*	Serine/threonine kinase expressed bipolar cell subsets	[36,37]
Rhodopsin	*RHO*	Visual pigment protein expressed in rod photoreceptors	[38]
Recoverin	*RCVRN*	Calcium-binding protein expressed in rod and cone photoreceptors	[39]
Retinaldehyde binding protein 1	*RLBP1*	Visual cycle protein expressed in Müller cells and retinal pigment epithelial cells	[40,41]
Retinoid isomerohydrolase RPE65	*RPE65*	Visual cycle protein expressed in retinal pigment epithelial cells	[42]
**Parasite Transcripts**			
Dense granule protein 6	*GRA6*	*T. gondii* virulence factor and activator of host NFAT4	[43]
Rhoptry protein 16	*ROP16*	*T. gondii* virulence factor and activator of host STAT3	[44,45]
Surface antigen 1	*SAG1*	*T. gondii* host cell attachment and invasion protein	[46,47]

**Table 2:** Sequences and amplicon sizes of primer pairs used in quantitative polymerase chain reactions.

Gene Transcript	Primer Sequences	Size (bp)	NCBI Accession Number
*CCL2* ^†^	FWD: 5′-GCATGAAAGTCTCTGCCGC-3′ REV: 5′-GGACACTTGCTGCTGGTGATT-3′	181	NM_002982.4
*CXCL10* ^†^	FWD: 5′-CCTATCTTTCTGACTCTAAGTGGC-3′ REV: 5′-ACGTGGACAAAATTGGCTTG-3′	148	NM_001565.4
*CXCL8* ^†^	FWD: 5′-CCAGGAAGAAACCACCGGAA-3′ REV: 5′-TCTCAGCCCTCTTCAAAAACTTC-3′	336	NM_000584.4
*GAD2* [48]	FWD: 5′-GCGTGGAGAGGGCCAACTCTG-3′ REV: 5′-CCCGGTAGTCCCCTTTGCCC-3′	251	NM_000818.3
*GRA6* ^†^	FWD: 5′-AAGCAGACCCCTTCGGAAAC-3′ REV: 5′-TCTTCAGCTAACGAGTCGCC-3′	119	-
*IL6* [49]	FWD: 5′-ATGCAATAACCACCCCTGACC-3′ REV: 5′-CCATGCTACATTTGCCGAAGAG-3′	160	NM_000600.5
*LHX1* [50]	FWD: 5′-ATGCAACCTGACCGAGAAGT-3′ REV: 5′-CAGGTCGCTAGGGGAGATG-3′	121	NM_005568.5
*MKI67* [51]	FWD: 5′-TGACCCTGATGAGAAAGCTCAA-3′ REV: 5′-CCCTGAGCAACACTGTCTTTT-3′	141	NM_002417.5
*OTX2* [52]	FWD: 5′-CATGAGGCTGTAAGTTCCAC-3′ REV: 5′-TTGTTTGGAGGTGCAAAGTC-3′	126	NM_021728.4
*PAX6* [53]	FWD: 5′-TAAGGATGTTGAACGGGCAG-3′ REV: 5′-TGGTATTCTCTCCCCCTCCT-3′	126	NM_000280.6
*PRKCA* [52]	FWD: 5′-CCCGACACTGATGACCCCA-3′ REV: 5′-AAGTCCATAGAGCAGTGACCC-3′	99	NM_002737.3
*POU4F1* [54]	FWD: 5′-TTTCCTCCACCCATTCTCTG-3′ REV: 5′-ACCCCAGTCCTCAAGGCTA-3′	62	NM_006237.4
*RCVRN* [52]	FWD: 5′-GGGACCATCAGCAAGAAT-3′ REV: 5′-GATCTTCTCGGCTCGCTTT-3′	123	NM_002903.3
*RHO* [52]	FWD: 5′-GTCCAGGTACATCCCCG-3′ REV: 5′-ACGAACATGTAGATGACAA-3′	102	NM_000539.3
*RLBP1* ^†^	FWD: 5′-CCTCTCTAGTCGGGACAAGTAT-3′ REV: 5′-TCATCTCAAGCAGCCCTTTC-3′	607	NM_000326.4
*ROP16* ^†^	FWD: 5′-GAGCCAATACGGTCTGCCAT-3′ REV: 5′-CACAGCTCGCTGGAAGAGAA-3′	187	-
*RPE65* ^†^	FWD: 5′-CTGGAGCCTGAAGTTCTCTTT-3′ REV: 5′-AAGATGGGTTCTGATGGGTATG-3′	209	NM_000329.2
*RPLP0* [55]	FWD: 5′-GCAGCATCTACAACCCTGAA-3′ REV: 5′-GCAGATGGATCAGCCAAGAA-3′	235	NM_053275.3
*SAG1* [56]	FWD: 5′-GCTGTAACATTGAGCTCCTTGASTTCCTG-3′ REV: 5′-CCGGAACAGTACTGATTGTTGTCTTGAG-3′	350	-
*VSX2* [53]	FWD: 5′-ACCAGACCAAGAAACGGAAG-3′ REV: 5′-GGGTAGTGGGCTTCGTTG-3′	97	NM_182894.3
*YWHAZ* [57]	FWD: 5′-ACTTTTGGTACATTGTGGCTTCAA-3′ REV: 5′-CCGCCAGGACAAACCAGTAT-3′	98	NM_003406.4

Abbreviations: *CCL2* = C-C motif chemokine ligand 2, *CXCL10* = C-X-C motif chemokine ligand 10, *CXCL8* = C-X-C motif chemokine ligand 8, *GAD2* = glutamate decarboxylase 2, *GRA6* = *T. gondii* dense granule protein 6, *IL6* = interleukin-6, *LHX1* = LIM homeobox 1, *MKI67* = marker of proliferation Ki-67, *OTX2* = orthodenticle homeobox 2, *PAX6* = paired box 6, *PRKCA* = protein kinase c alpha, *POU4F1* = POU class 4 homeobox 1, *RCVRN* = recoverin, *RHO* = rhodopsin, *RLBP1* = retinaldehyde binding protein 1, *ROP16* = *T. gondii* rhoptry protein 16, *RPE65* = retinoid isomerohydrolase RPE65, *RPLP0* = ribosomal protein lateral stalk protein subunit P0, *SAG1* = *T. gondii* surface antigen 1, *VSX2* = visual system homeobox 2, and *YWHAZ* = tyrosine 3-monooxygenase/tryptophan 5-monooxygenase activation protein zeta. ^†^ Primers designed in-house, and amplicons confirmed by sequencing.

## Data Availability

All data are contained within the article or the Supplementary Materials.

## Supplementary Materials

The following supporting information can be downloaded at: https://www.mdpi.com/article/10.3390/pathogens14030286/s1, Figure S1: Proliferation, multipotency or retinal cell-specific gene transcripts in human retinal organoids expressed as fold-change; Figure S2: *Toxoplasma gondii* tachyzoite infection of human retinal organoids imaged at low power magnification; Figure S3: *Toxoplasma gondii* tachyzoite infection of human retinal organoids visualized to show parasite rosettes.

## Abbreviations

The following abbreviations are used in this manuscript:

ESC	Embryonic stem cell
iPSC	Induced pluripotent stem cell
RT-qPCR	Reverse transcription-quantitative polymerase chain reaction
DMEM	Dulbecco’s Modified Eagle Medium
FBS	Fetal bovine serum
FGF	Fibroblast growth factor
AUC	Area under the curve
IgG	Immunoglobulin
CCL2	C-C motif chemokine ligand 2
CXCL10	C-X-C motif chemokine ligand 10
CXCL8	C-X-C motif chemokine ligand 8
GAD2	Glutamate decarboxylase 2
GRA6	*T. gondii* dense granule protein 6
IL6	Interleukin 6
LHX1	LIM homeobox 1
MKI67	Marker of proliferation Ki-67
OTX2	Orthodenticle homeobox 2
PAX6	Paired box 6
PRKCA	Protein kinase c alpha
POU4F1	POU class 4 homeobox 1
RCVRN	Recoverin
RHO	Rhodopsin
RLBP1	Retinaldehyde binding protein 1
ROP16	*T. gondii* rhoptry protein 16
RPE65	Retinoid isomerohydrolase RPE65
RPLP0	Ribosomal protein lateral stalk protein subunit P0
SAG1	*T. gondii* surface antigen 1
VSX2	Visual system homeobox 2
YWHAZ	Tyrosine 3-monooxygenase/tryptophan 5-monooxygenase activation protein zeta
