# Supplementary material for: Human Retinal Organoid Model of Ocular Toxoplasmosis
Source: Pathogens. 2025 Mar 14;14(3):286. doi: 10.3390/pathogens14030286 (PMC11945118; doi:10.3390/pathogens14030286)
Supplement: Supplementary file 1 [file pathogens-14-00286-s001.zip › pathogens-3496051-supplementary.pdf]

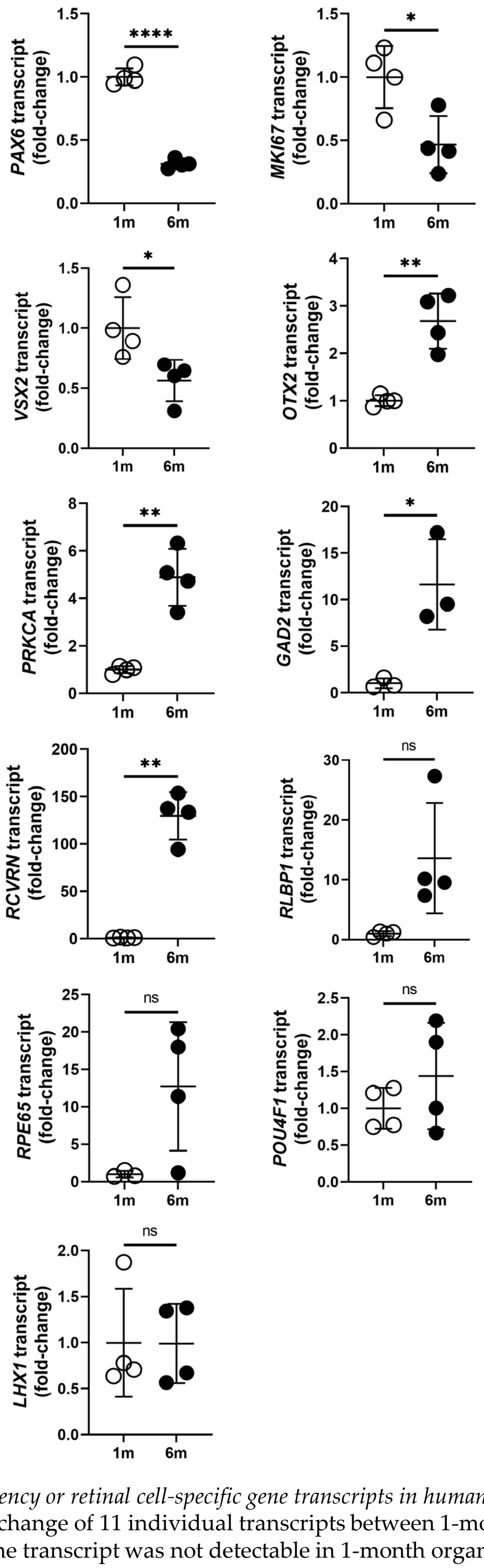

Figure S1. *Proliferation, multipotency or retinal cell-specific gene transcripts in human retinal organoids expressed as fold-change.* Graphs presenting fold-change of 11 individual transcripts between 1-month and 6-month (m) organoids: no graph is provided for *RHO* as the transcript was not detectable in 1-month organoids. In all graphs, open and closed circles represent gene transcript expression in 1-month and 6-month organoids, respectively (n = 4 organoids/time interval, cultured in different wells of 12-well plates). Transcript fold-changes were compared by unpaired two-tailed t-test with Welch's correction: cross bar = mean expression; error bar = standard deviation; ns = not significant; * = $p<0.05$; ** = $p<0.01$; *** = $p<0.001$; **** = $p<0.0001$. Labels: *PAX6* = paired box 6, *MKI67* = marker of proliferation Ki-67, *VSX2* = visual system homeobox 2, *OTX2* = orthodenticle homeobox 2, *POU4F1* = POU class 4 homeobox 1, *LHX1* = LIM homeobox 1, *GAD2* = glutamate decarboxylase 2, *PRKCA* = protein kinase c alpha, *RHO* = rhodopsin, *RCVRN* = recoverin, *RLBP1* = retinaldehyde binding protein 1, *RPE65* = retinoid isomerohydrolase RPE65.

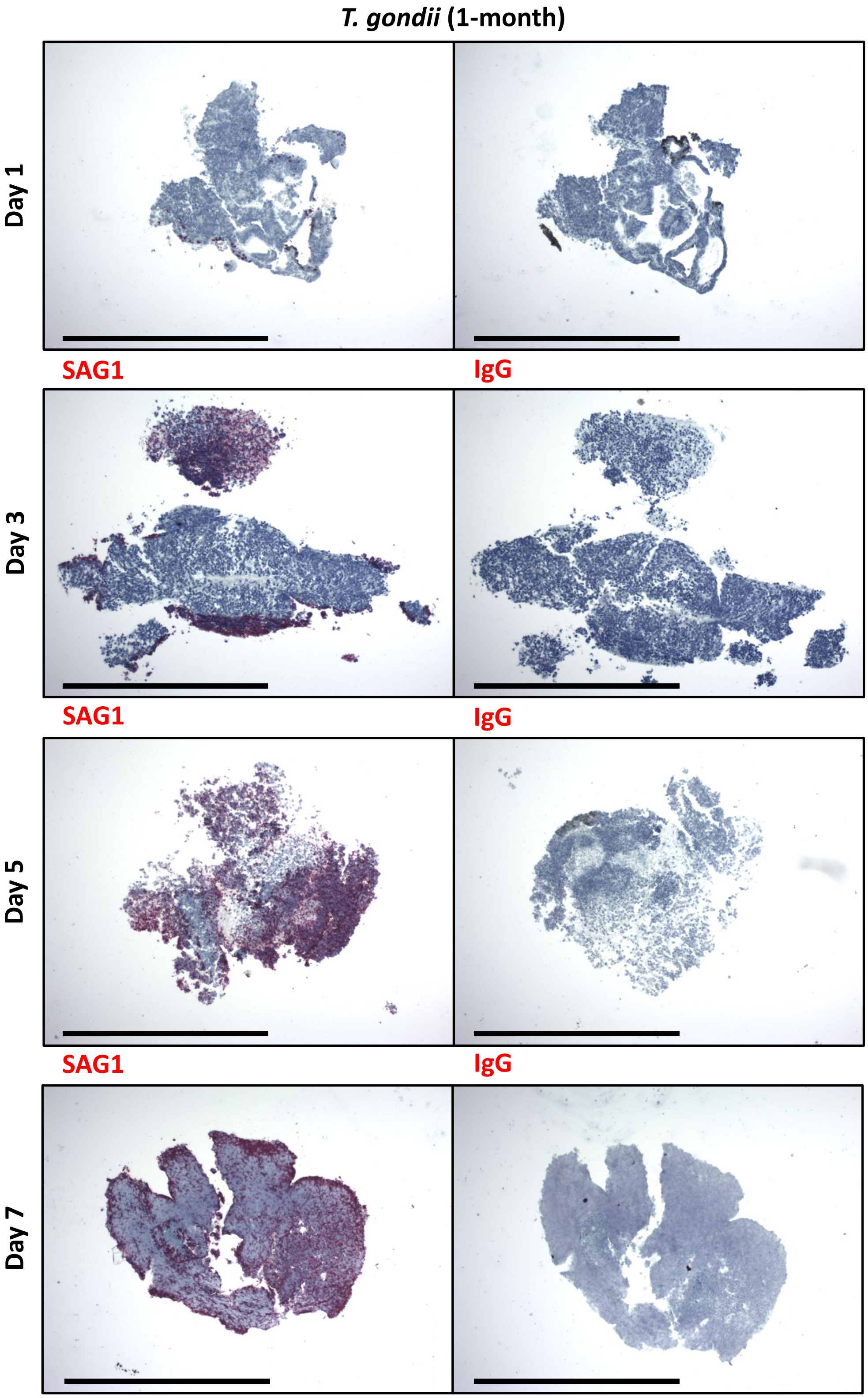

Figure S2. *Toxoplasma gondii tachyzoite infection of human retinal organoids imaged at low power magnification.* Photomicrographs of 1-month retinal organoids immunolabeled for *T. gondii* surface antigen 1 (SAG1) or species- and isotype-matched immunoglobulin (IgG), taken up to 7 days following incubation with live tachyzoites. Fast Red chromogen (red) with hematoxylin nuclear counterstain (blue). Original magnification 100X. Scale bar = 500 microns.

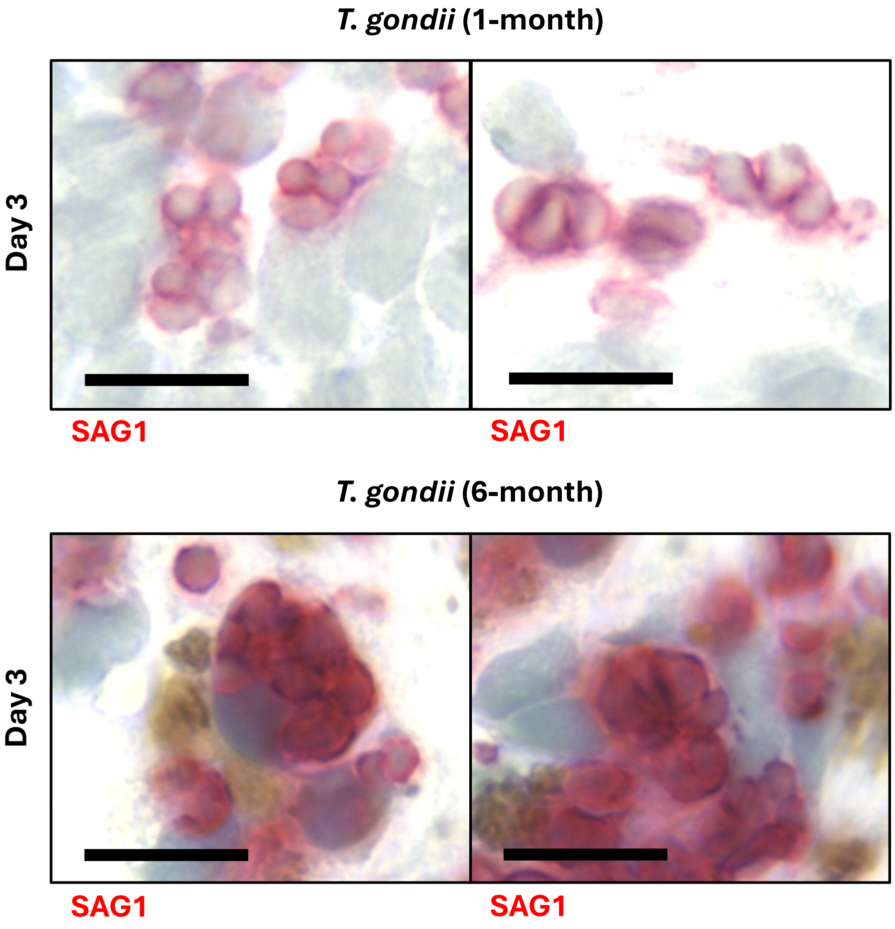

Figure S3. *Toxoplasma gondii tachyzoite infection of human retinal organoids visualized to show parasite rosettes.* Enlarged sections of photomicrographs of 1-month and 6-month retinal organoids immunolabeled for *T. gondii* surface antigen 1 (SAG1), taken 3 days following incubation with live tachyzoites. Fast Red chromogen (red) with hematoxylin nuclear counterstain (blue). Original magnification = 1000X. Scale bar = 10 microns.
